# Small Nuclear Ribonucleoprotein Polypeptide N Accelerates Malignant Progression and Poor Prognosis in Colorectal Cancer Transcriptionally Regulated by E2F8

**DOI:** 10.3389/fonc.2020.561287

**Published:** 2020-11-02

**Authors:** Meiling Ji, Li Ren, Yang Lv, Xinyuan Lao, Qingyang Feng, Wentao Tang, Aobo Zhuang, Tianyu Liu, Peng Zheng, Jianmin Xu

**Affiliations:** Department of General Surgery, Zhongshan Hospital Fudan University, Shanghai, China

**Keywords:** colorectal cancer, SNRPN, proliferation, metastasis, E2F8

## Abstract

Colorectal cancer is a major cause of death worldwide, and the identification of new diagnostic and prognostic biomarkers is crucial to develop new strategies to avoid colorectal cancer-related deaths. Small nuclear ribonucleoprotein polypeptide N (SNRPN) is an imprinted gene that plays an important role in various neurodevelopmental disabilities. In this study, SNRPN was highly expressed in colorectal cancer tissues and involved in the progression of this disease. Immunohistochemistry analysis of 1,310 colorectal cancer tissue samples showed that SNRPN highly expressed in cancer tissues than in adjacent tissues and was mainly localized in the nucleus. Clinical pathological factor analysis demonstrated that higher expression of SNRPN was significantly associated with larger tumor size, location of the tumor on the left-sided colon, neural invasion, and distant metastasis. Univariate and multivariate analyses showed that SNRPN expression was an independent risk factor for survival, with high expression levels indicating worse overall survival. Both *in vitro* and *in vivo* experiments confirmed that high expression of SNRPN was associated with tumor proliferation, cell cycle, and metastasis. Knocking down SNRPN blocked the cell cycle at the G2/M phase transition and promoted tumor cell apoptosis, inhibiting the progression of colorectal cancer. To explore the up-steam of SNRPN, we found by luciferase reporter assay and chromosomal immunoprecipitation assay that E2F8 was a transcriptional regulator up-steam of SNRPN in colorectal cancer. Systematic studies of SNRPN will help us discover new regulatory molecules and provide a theoretical basis for finding new molecular targets for this disease.

## Introduction

Colorectal cancer (CRC) is one of three most common cancers both in the incidence and the mortality worldwide (1). As a gene-driven cancer, uncovering the molecular cascade from adenoma to cancer has greatly promoted the understanding of CRC tumorigenesis and development. Recently, with the use of targeted drugs and surgical treatment, the prognosis of the CRC patient has largely improved. However, the benefits to the overall population of patients with CRC are limited. To discover new molecular aspects and further explore the underlying molecular mechanisms are still important methods to improve early detection and prevention and can promote the development of innovative therapeutic strategies.

The small nuclear ribonucleoprotein polypeptide N (SNRPN) is an imprinted gene. It has been linked to various neurodevelopmental disorders in previous researches, for example, Angelman syndrome (AS), Prader–Willi syndrome (PWS), and autism spectrum disorders (ASDs) (2, 3). Li et al. demonstrated that the abnormal expression of SNRPN impairs neurological function through regulating the nuclear receptor subfamily, group A, member 1(Nr4a1) (4). SNRPN encodes an RNA-binding (SmN and Sm51) protein, SNRPN, which involved in pre-mRNA splicing, and it is one member of the small nuclear ribonucleic particle SMB/SMN family. SNRPN is responsible for splicing the calcitonin/CGRP transcript in the brain (5), and the alternative splicing also has been demonstrated due to the reduced expression levels of mature U2 snRNP. The expression of SNRPN is tissue specific, and the highest expression in adult is found in the heart and brain (6).

SNRPN is also known to be related to the onset of obesity, and the lack of SNRPN expression results in hyperphagia, loss of satiety, and obesity in PWS (7, 8). David et al. demonstrated that combination of three single-nucleotide polymorphisms (SNPs), rs12905653, rs1391516, and rs2047433, can predict the probability of obesity much better from a case–control study with a sample of 265 Spanish patients (9). SNRPN also plays a role in regulating osteoblastic differentiation of bone marrow-derived mesenchymal stem cells (BM-MSCs) by increasing runt-related transcription factor 2 (Runx2) expression at the RNA splicing level (10).

There are also some researches in cancers. Inactivation of the SNRPN gene results in lung and brain cancer (11, 12). Deletions of the SNRPN locus were discovered in gliomas and glioblastoma, selected as the down-regulated expressed gene (13). Increased CpG methylation was found in gastric cancer vs. non-metaplastic mucosa and showed a significant decreased expression in gastric cancer (14). SNRPN is a key player in pancreatic adenocarcinoma and medulloblastoma cell growth (15, 16). However, the role of SNRPN in CRC remains unknown. In this article, we investigated SNRPN expression levels in the tissues of CRC patients and their association with clinical prognosis. We also investigated the participation of SNRPN in the progression of CRC *in vitro* and *in vivo*, as well as the mechanism involved.

## Materials and Methods

### Study Patients

A total of 1,310 consecutive CRC patients in Zhongshan Hospital Fudan University were recruited in the study retrospectively. The patients underwent radical primary tumor resections without prior treatment from 2008 to 2012. The stages of cancer were determined referring to the 8th edition of the International Union Against Cancer (UICC)/American Joint Committee on Cancer (AJCC) TNM classification. The median follow-up time was 50.5 months. The ethical approval was given by the Clinical Research Ethics Committee of Zhongshan Hospital, Fudan University. Informed consent for the acquisition of tissue samples and clinical data was obtained from all patients.

### Immunohistochemistry

Standard procedures were used to determine the levels of SNRPN expression in CRC tumor samples. After being dried overnight at 37°C and deparaffinized in xylene, the tissue microarray (TMA) slide was rehydrated through graded alcohol and then immersed in 3% hydrogen peroxide to block endogenous peroxidase activity. After that, it was antigen-retrieved with microwave heating. Then, slides were incubated with 10% normal goat serum at room temperature to reduce nonspecific reactions. The primary anti-SNRPN antibody produced in rabbit (Sigma-Aldrich Cat# HPA003482, RRID: AB_1857337) and primary anti-E2F8 antibody produced in rabbit (LS bio cat# RRID: LS-C804191) were diluted (1:100) in 3% bovine serum albumin (BSA) with 1× phosphate-buffered saline (PBS) and incubated overnight. They were sequentially incubated with a polymer peroxidase-labeled secondary antibody at room temperature and then stained with 3,3′-diaminobenzidine (DAB).

### Evaluation of Immunohistochemistry

The staining index of SNRPN was determined by multiplying the score of staining intensity with staining proportion. The score of intensity are as follows: 0 = negative, 1 = weak positive, 2 = moderate positive, and 3 = strong positive. The area score was the percentage of positive cells among all tumor cells multiplied by 100. Finally, the intensity score was multiplied by the area score, ranging from 0 to 300. If the staining of sample was heterogeneous, each component region was scored independently and then summed. The immunohistochemistry (IHC) score was assessed by two pathologists independently.

### Lentivirus Packaging and Transduction

The siRNA sequence targeting human SNRPN gene (NM_003097.5) was designed as follows: S1-GATCCGAATCTTCATTGGCACCTTTACTCGAGTAAAGGTGCCAATGAAGATTCTTTTTG and S2-GATCCGTTCAGAAAGATCAAGCCAAACTCGAGTTTGGCTTGATCTTTCTGAACTTTTTG. Both siRNAs were inserted into the vector pGp (SBI, USA), and the restriction enzyme sites were BamHI and EcoRI. HEK293T cells was transfected with the siRNA-harboring plasmid and two helper plasmids, pVSVG-I and pCMVΔR8.92 (TIANGEN, China). Two days later, the cell culture media was obtained and then concentrated to get the recombinant lentivirus. The multiplicity of infection (MOI) of the recombinant lentivirus-containing media was 15 when added to the HCT116 cells.

### Quantitative Real-Time PCR

Total cellular RNA was extracted through TRIzol reagent method (Life Technologies, USA). Then, RNA was reverse transcribed by PrimeScript RT Reagent Kit (TaKaRa, Japan). Real-time PCR was performed using SYBR master mixture (TaKaRa, Japan) on the Bio-Rad Connect Real-Time PCR platform. The primer sequences of human SNRPN are as follows: 5′- GTTTTGGGTCTGGTGTTGCT−3′ (forward) and 5′- TCATTACCTGC TGGGA TGGT−3′ (reverse). Comparative cycle threshold methods were used to calculate the relative quantities of mRNA normalized by ACTIN.

### MTT Assay

Lentivirus-infected SW1116 and HCT116 cells were seeded in 96-well plates at an inoculation density of 2,500 and 3,000 cells/well, respectively. MTT (3-(4, 5-dimethylthiazol-2-yl)-2, 5-diphenyltetrazolium bromide) solution was added when incubated on days 1 to 5. The medium was then removed and acidic isopropanol (10% sodium dodecyl sulfate (SDS), 5% isopropanol, and 0.01 mol/l HCl) was added. Plates were read at a wavelength of 595 nm.

### Colony Formation Assay

After lentivirus infection, SW1116 and HCT116 cells were seeded in six-well plates at a density of 200 cells/well. The medium was changed at 3-day intervals. After 8 days in 5% CO2 incubator at 37°C, cells were fixed by4 % paraformaldehyde and then stained with crystal violet solution.

### Transwell Assay

After being trypsinized, cells were suspended in low serum media containing 0.1% fetal bovine serum (FBS) and seeded at 1 × 10^4^ with 100 μl on the upper chamber, and 500 μl media with 10% FBS (BD Biosciences, USA) was added to the lower chamber. After being cultured for 48 h, the cells on the apical side of each insert were removed and washed with PBS. The invading cells were fixed with 4% paraformaldehyde for 30 min and stained by crystal violet. Invasion ability was assayed using the transwell experiment pre-cultured with Matrigel.

### Cell Cycle Assay

SW1116 cells were harvested and fixed in 75% ethanol at 4°C overnight and washed by PBS before resuspension in RNase A/propidium iodide solutions (Beyotime Biotechnology, China). After being incubated for 1 h at room temperature, stained cells were analyzed by flow cytometer (Beckman, Gallios, USA).

### Apoptosis Assay

Cell apoptosis was assessed using an Annexin V/7-AAD double staining kit (KeyGen Biotech, China). Cells were harvested, proceeded following the manufacturer's protocol, and analyzed using a flow cytometer (Beckman, Gallios, USA).

### Western Blotting

Cells were collected and lysed in 2× SDS sample buffer (100 mM Tris-HCl, pH 6.8; 10 mM EDTA; 4% SDS; and 10% glycine). Samples containing 30 μg protein ran on SDS-PAGE gel at 80 V. Proteins were then transferred to PVDF membrane at 300 mA for 90 min. After being blocked by 1% BSA in TBS-T for 1 h at room temperature, the membrane was incubated with the primary antibodies overnight at 4°C. After that, membranes were incubated with secondary antibodies at room temperature. The signals were determined by the enhanced chemiluminescence kit (Pierce, USA).

### *In vivo* Tumorigenic Assay

Mice were injected subcutaneously with 8 × 10^6^ SW1116 cells down-regulated SNRPN by lentivirus and its control group to establish a CRC tumorigenicity model. Animal experiments were performed with conformity to the guidelines for use of experimental animals. The male mice were obtained from the Shanghai SLAC Laboratory Animal Co. Ltd. Mice were sacrificed after 19 days, and subcutaneous tumors were collected. Tumor size was estimated by the maximum diameter of the tumor (cm).

### Luciferase Reporter Assay

E2F8 regulation on the activity of SNRPN promoter was determined by dual-luciferase reporter assay. Briefly, the promoter binding region of SNRPN was cloned in the pGL3-Promoter Vector between Nhe I and Hind III downstream of the luciferase reporter. E2F8 was conducted in PEGFP-N1 vector between Hind III and BamH I. The vectors were co-transfected into HEK293T cells. At 48 h after transfection, we harvested the cells and measured the relative luciferase activity using Dual Luciferase Reporter assay system (Promega,USA). The luciferase activity was measured per 1,000 cells. The firefly luciferase activity/Renilla luciferase activity for each construct was compared with the pGL3-Basic vector control. All experiments were performed at least three times.

### Chromatin Immunoprecipitation Assays

Chromatin immunoprecipitation (ChIP) was conducted as described on manual. Briefly, HEK293T cells were harvested and cross-linked with 1% formaldehyde and quenching was performed with glycine. After being sonicated into 200–1,000 bp fragments, protein G agarose was added to the complex (antibody and chromatin), and the mixture was incubated at 4°C overnight. E2F8 antibody (Abcam Cat# ab109596) was added to pull down DNA from formaldehyde cross-linked chromatin. Then, protein G agarose antibody/chromatin complexes were resuspended by wash buffer and centrifuged for collection. The protein/DNA complexes were cleaved to get free DNA. Finally, the purified DNA was quantified using qPCR and the primers are as follows:

SNRPN-F1:TGCTGGGATTACAAGTGTGAG;

SNRPN-R1:GCAAGGACTATGAACAGC;

SNRPN-F2:GTGCTGGGATTACAAGTGTG;

SNRPN-R2:AGCAAGGACTATGGAGACAG.

### Statistical Analysis

SPSS statistical package was used to analyze clinical data. E2F8 and SNRPN expression between normal and cancer tissues was compared by paired Wilcoxon signed-rank test. The correlations between continuous valuables were analyzed using Spearman rank correlation test and χ^2^ test. Kaplan–Meier method was carried out to analyze OS, and the results were compared by log-rank test. Hazard ratio [HR, 95% confidence interval (CI)] was conducted to express risk factors. Statistical significance was defined as a *P* value <0.05. Three independent experiments were conducted for statistical analysis and *t* test was performed.

## Results

### SNRPN Is Highly Expressed in CRC Tumor Tissues

**Figure 1:**
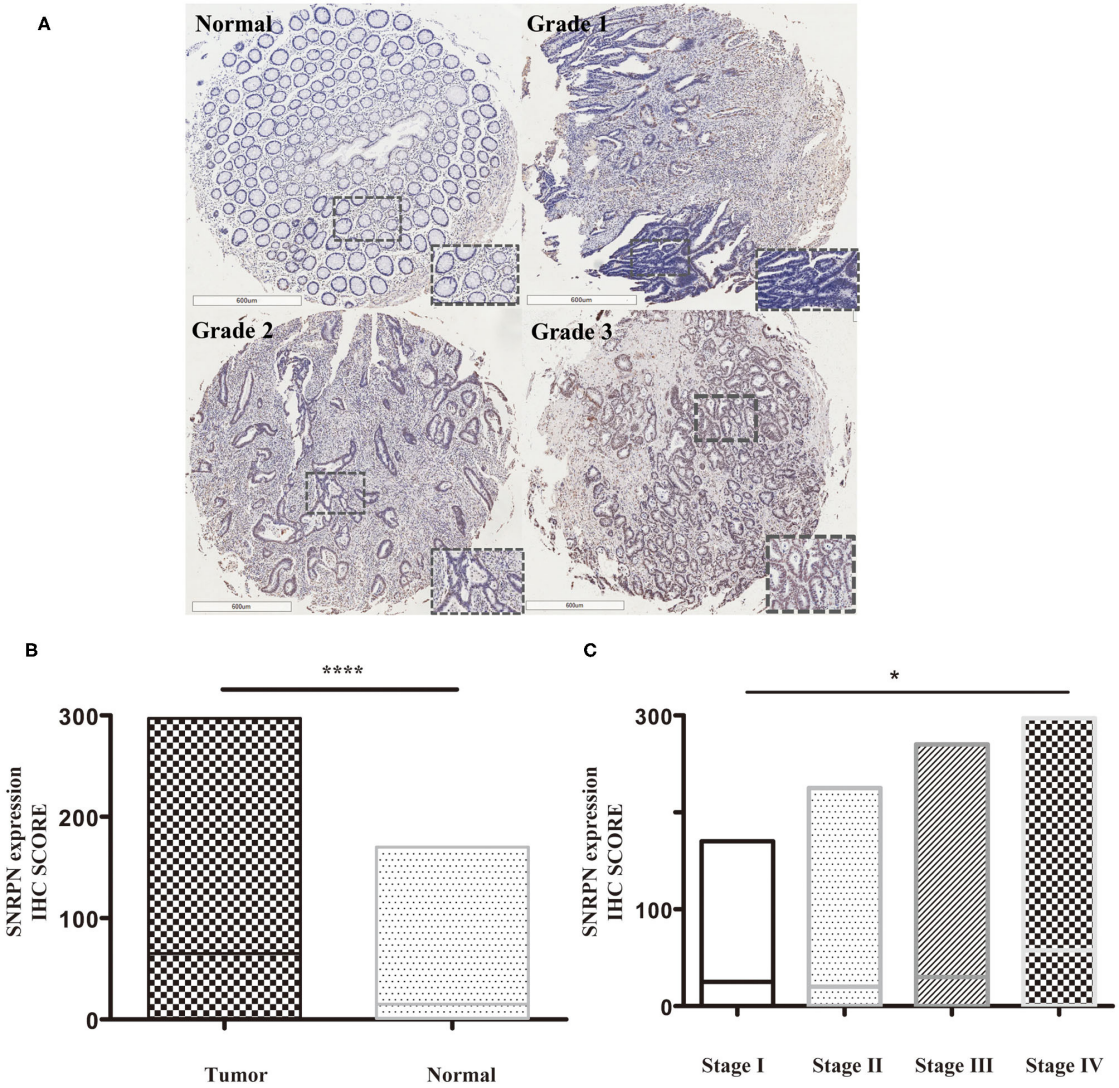
SNRPN is highly expressed in colorectal cancer tumor tissues. Immunohistochemistry (IHC) staining of SNRPN in colorectal cancer tissues. **(A)** Representative images of IHC staining intensity: normal—no staining, grade 1—weak staining, grade 2—moderate staining, and grade 3—strong staining. **(B)** IHC staining score of SNRPN was significantly higher in tumor tissues than in normal tissues. **(C)** IHC staining score of SNRPN in four stages. ^*^P < 0.05; ^****^P < 0.001. *P* value: Wilcoxon signed rank test (matched pairs).

Expression levels of SNRPN were determined using IHC staining. The results show that the subcellular localization of SNRPN is in the nucleus and that the positive expression rate of SNRPN was 89.31%. The scores of IHC in tumor tissues were significantly higher than in normal tissues (*P* < 0.001), suggesting the accumulated expression of SNRPN in CRC tissues (Figure 1B). Representative pictures of different expression patterns of SNRPN are shown in Figure 1A. As CRC staging progressed, SNRPN expression increased: significantly different expression levels were observed when comparing stage IV with I (Figure 1C).

### The Association Between SNRPN Expression Levels and Clinical Characteristics

In the study, samples with IHC scores between 5 and 9 were considered as high SNRPN expression, and IHC scores between 0 and 4 were considered as low expression. SNRPN was highly expressed in 427 samples and lowly expressed in 883 samples.

The association between SNRPN expression levels and the clinic-pathological characteristics of patients is shown in Table 1. Analysis demonstrates that higher expression of SNRPN was significantly associated with larger tumor size (*P* = 0.000), left-sided colon (*P* = 0.008), neural invasion (*P* = 0.000), and distant metastasis (*P* = 0.030).

**Table 1:** Relationship between SNRPN and clinical characteristics in included CRC patients.

**Factors**	**Total**	**SNRPN expression**		
		**Low**	**High**	***P* value**
All patients	1,310	883	427	
Age (years)				0.325
≥60	728 (55.6%)	499 (56.6%)	229 (53.6%)	
<60	582 (44.4%)	384 (43.4%)	198 (46.4%)	
Gender				0.365
Male	787 (60.6%)	538 (60.8%)	249 (58.3%)	
Female	523 (40.0%)	345 (39.2%)	178 (41.7%)	
CEA (ng/ml)				0.628
≤5	689 (52.6%)	469 (53.2%)	220 (51.5%)	
>5	591 (45.1%)	392 (44.4%)	199 (46.6%)	
Unknown	30 (2.3%)	22 (2.4%)	8 (1.9%)	
Tumor size (cm)				**0.000**
≤5	489 (37.3%)	368 (41.7%)	121 (29.0%)	
>5	821 (62.7%)	515 (58.3%)	306 (71.0%)	
Tumor differentiation				0.287
Well/moderate	894 (68.2%)	611 (69.18%)	283 (66.3%)	
Poor/anaplastic	416 (31.8%)	272 (30.82%)	144 (33.7%)	
Pathological type				0.329
Mucinous	173 (13.2%)	111 (12.6%)	62 (14.5%)	
Non-mucinous	1,137 (86.8%)	772 (87.4%)	365 (85.5%)	
Vascular invasion				0.904
No	1,122 (85.6%)	757 (85.7%)	365 (85.5%)	
Yes	188 (14.4%)	126 (14.3%)	62 (14.5%)	
Neural invasion				**0.000**
No	1,214	855 (96.8%)	359 (84.1%)	
Yes	96	28 (3.2%)	68 (15.9)	
Tumor location				**0.008**
Right-sided colon	364 (27.8%)	265 (30.0%)	99 (23.19%)	
Left-sided colon	341 (26.0%)	211 (23.9%)	130 (30.44%)	
Rectum	605 (46.2%)	407 (46.1%)	198 (46.37%)	
T stage				0.421
T1/T2	237 (18.1%)	165 (18.7%)	72 (16.9%)	
T3/T4	1,073 (81.9%)	718 (81.3%)	355 (83.1%)	
N stage				0.966
N0	719 (54.9%)	485 (54.9%)	234 (54.8%)	
N1/N2	591 (45.1%)	398 (45.1%)	193 (45.2%)	
M stage				**0.030**
M0	1,005 (76.7%)	693 (78.5%)	312 (73.1%)	
M1	305 (23.3%)	190 (21.5%)	115 (26.9%)	

*Bold emphasis has showed the significance of the values*.

### Higher Expression Levels of SNRPN Indicate Poorer Survival in CRC

**Figure 2:**
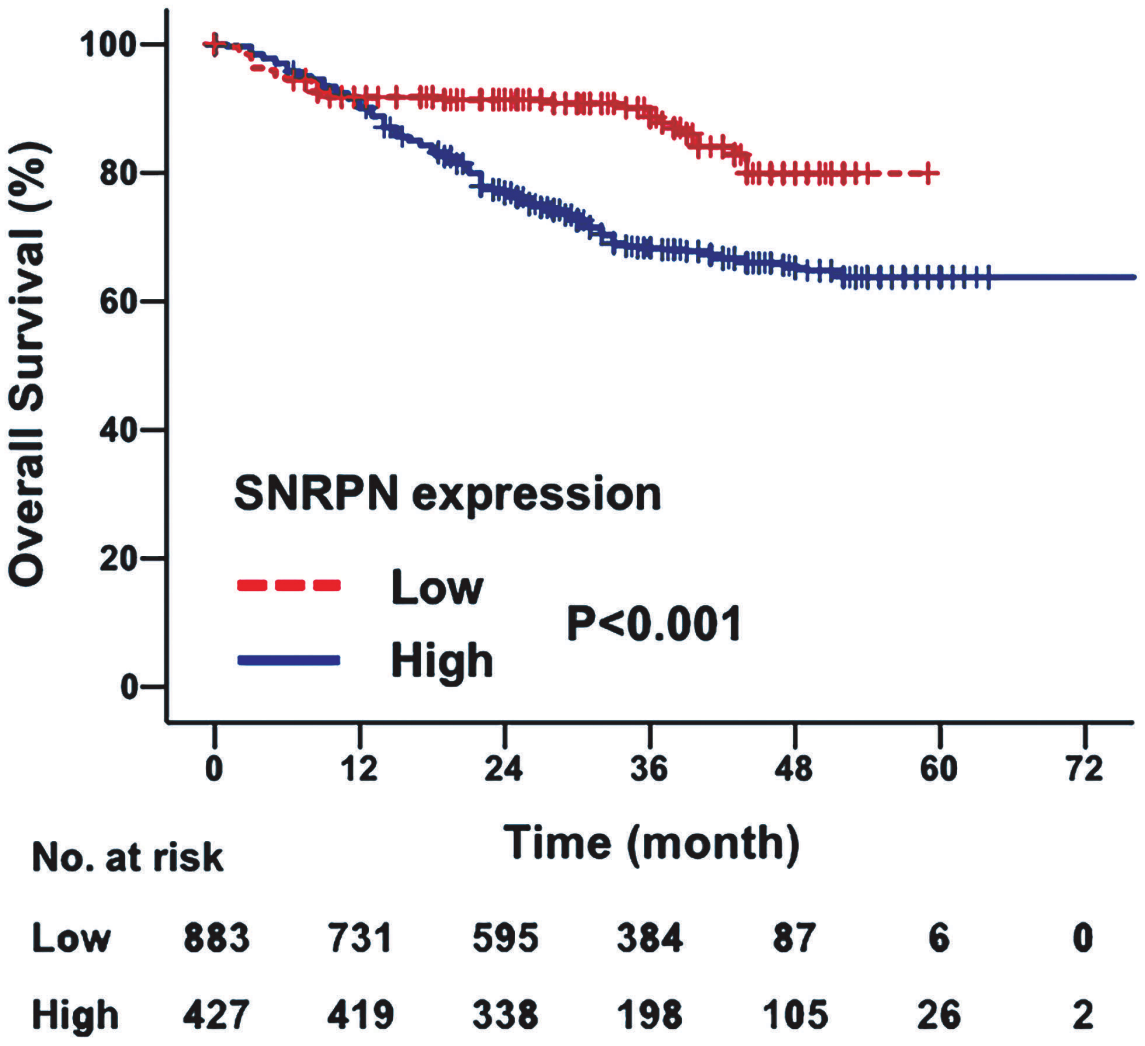
Downregulation of SNRPN in tumor tissues indicates poor overall survival. Kaplan–Meier survival curve with expression levels of SNRPN in tumor tissues for colorectal cancer patients after primary tumor resection. HR, hazard ratio; CI, confidence interval; *P* value, log-rank test.

The median follow-up time for all patients was 31.0 months (ranging from 2 to 97 months). The overall survival of patients with highly expressed SNRPN was significantly worse than that of lowly expressed patients (HR = 2.086, 95% CI = [1.749–2.446], *P* < 0.001). The 3- and 5-year OS rates were 67.3 and 62.2% for patients with high expression of SNRPN, respectively. For patients with low expression of SNRPN, the 3- and 5-year OS rates were 87.9 and 82.1 % (Figure 2). These results suggest that high expression of SNRPN predicts worse OS.

To investigate the risk factors affecting the prognosis of CRC, univariate and multivariate analyses of prognosis were performed. Univariate analysis shows that primary tumor differentiation (*P* = 0.009), T stage (*P* = 0.000), N stage (*P* = 0.000), M stage (*P* = 0.000), and SNRPN expression levels (*P* = 0.000) were associated with survival outcomes significantly. In addition, multivariate Cox regression analyses showed that SNRPN expression was an independent prognostic factor of OS in CRC patients (*P* = 0.000, HR = 2.030, 95% CI: 1.781–2.445) (Table 2).

**Table 2:** Univariate and multivariate Cox regression analyses for overall survival.

**Factors**	**Univariate analysis**		**Multivariate analysis**	
	**HR (95% CI)**	***P* value**	**HR (95% CI)**	***P* value**
Age level		0.159		
≥60	1 (reference)			
<60	1.111 (0.960–1.285)			
Gender		0.948		
Male	1 (reference)			
Female	0.995 (0.859–1.153)			
CEA (ng/l)		0.647		
≤5	1 (reference)			
>5	0.965 (0.831–1.122)			
Tumor location		0.699		
Right-sided colon	1 (reference)			
Left-sided colon	1.053 (0.883–1.255)			
Rectum	0.965 (0.806–1.156)			
Primary differentiation		0.009		0.875
Well/moderate	1 (reference)		1 (reference)	
Poor/anaplastic	1.243 (1.057–1.463)		0.988 (0.865–1.172)	
Tumor size (cm)		0.492		
≤5	1 (reference)			
>5	1.055 (0.905–1.230)			
Pathological type		0.263		
Mucinous	1 (reference)			
Non-mucinous	1.063 (0.956–1.181)			
Vascular invasion		0.141		
No	1 (reference)			
Yes	1.164 (0.951–1.424)			
Neural invasion		0.275		
No	1 (reference)			
Yes	1.024 (0.632–2.851)			
T stage		0.000		0.035
T1/T2	1 (reference)		1 (reference)	
T3/T4	1.941 (1.523–2.243)		1.232 (1.031–1.471)	
N stage		0.000		0.000
N0	1 (reference)		1 (reference)	
N1/N2	2.631 (2.125–3.048)		2.312 (2.010–2.678)	
M stage		0.000		0.000
M0	1 (reference)		1 (reference)	
M1	4.653 (3.891–5.563)		3.852 (3.135–4.560)	
SNRPN expression		0.000		0.000
Low	1 (reference)		1 (reference)	
High	2.086 (1.749–2.446)		2.030 (1.781–2.445)	

### Downregulation of SNRPN Suppresses CRC Cell Proliferation

**Figure 3:**
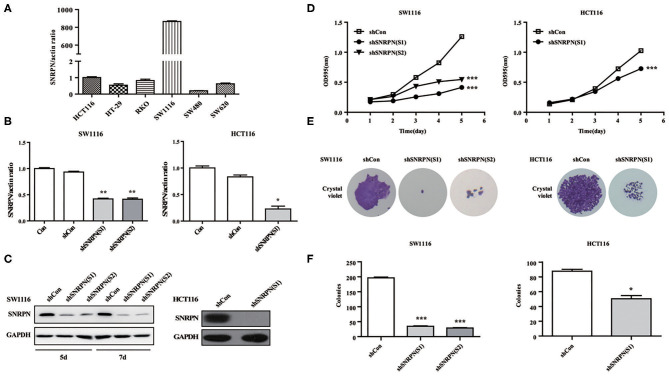
Downregulation of SNRPN suppresses malignant cell proliferation in colorectal cancer cell. **(A)** RT-PCR analysis of SNRPN mRNA levels in common colorectal cancer cell lines. **(B)** RT-PCR analysis of SNRPN mRNA levels after transduction with lentivirus containing shSNRPN(S1) and shSNRPN(S2) in SW1116 cells and shSNRPN(S2) in HCT116 cells. ^**^P < 0.01. **(C)** Western blotting analysis of SNRPN protein levels after transduction with lentivirus containing shSNRPN(S1) and shSNRPN(S2). **(D)** Growth curves of shCon, shSNRPN(S1), and shSNRPN(S2)-transduced cells determined using MTT assays. ^***^P < 0.001. **(E)** Representative images of colony formation assays. **(F)** Graph of colony numbers in the shCon, shSNRPN(S1), and shSNRPN(S2) groups. ^*^P < 0.05; ^***^P < 0.001.

To explore the biological function of SNRPN, we characterized it in cancer cells. Firstly, the basic expression of SNRPN in CRC cell lines was determined using qPCR, which shows higher expression levels in the SW1116 and HCT116 cell lines (Figure 3A). To further investigate the low expression of SNRPN in CRC, subsequent experiments employed these two cell lines. To eliminate off-target effects, we designed two shRNAs (S1 and S2) for different sequence segments of SNRPN and packed them into lentivirus. The results show that mRNA levels were effectively downregulated via shRNA(S1) and shRNA(S2) in SW1116 cells (Figure 3B). Western blotting results were consistent, and the downregulation of SNRPN in SW1116 cells was to some extent time dependent (Figure 3C). To explore the biological function of SNRPN in CRC, we examined cell proliferation using MTT and colony formation assays. MTT assay results show that the proliferation of SW1116 cells was significantly inhibited by downregulation of SNRPN (Figure 3D). In addition, colony formation experiments were consistent with these results (Figures 3E,F). Because shRNA(S1) had a stronger interference effect, it was utilized to transduce HCT116 cells for further experiments. shRNA(S1) could effectively downregulate the expression of SNRPN in HCT116 cell lines at both the mRNA and protein levels. We conclude that cell proliferation is inhibited after SNRPN downregulation.

### Downregulation of SNRPN Suppresses CRC Cell Metastasis

**Figure 4:**
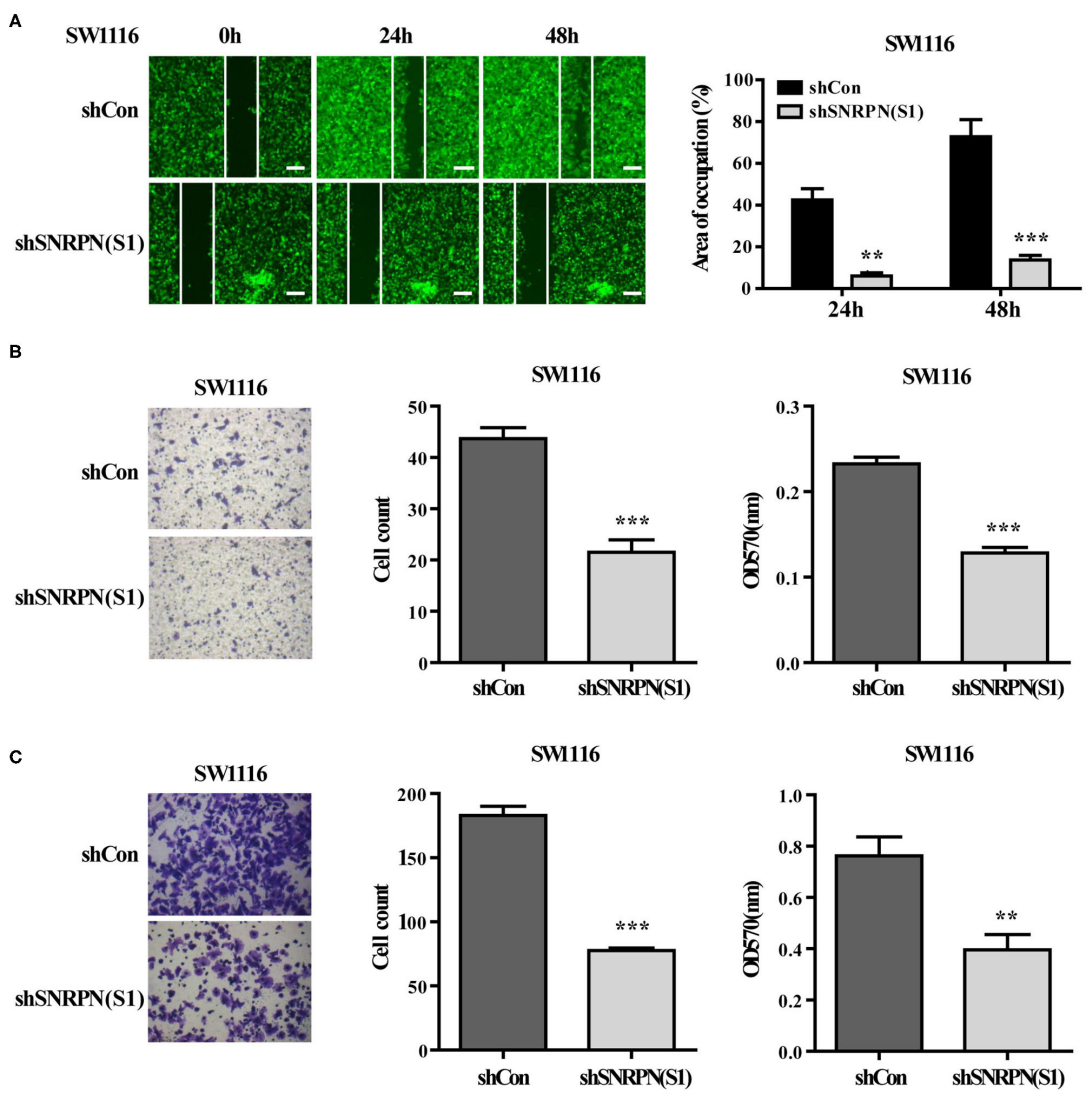
Downregulation of SNRPN suppresses metastasis in colorectal cancer cells. **(A)** Wound healing experiment assays showing migration of SW1116 cells after downregulation of SNRPN. Area of occupation shown by representative images and graphs. ^**^P < 0.01; ^***^P < 0.001. **(B)** Migration assay of SW1116 cells after downregulation of SNRPN using transwell experiments. ^***^P < 0.001. **(C)** Invasion assay of SW1116 cells after downregulation of SNRPN using transwell experiments with Matrigel. ^**^P < 0.01; ^***^P < 0.001.

The effects of SNRPN on metastasis was also examined using wound healing assays. The results show that, when SNRPN was downregulated, the ability to heal decreased (Figure 4A). In addition, transwell experiments indicate a decreased migration ability (Figure 4B), and transwell experiments with Matrigel show a reduced invasive ability (Figure 4C).

### SNRPN Inhibits the Cell Cycle and Promotes Apoptosis

**Figure 5:**
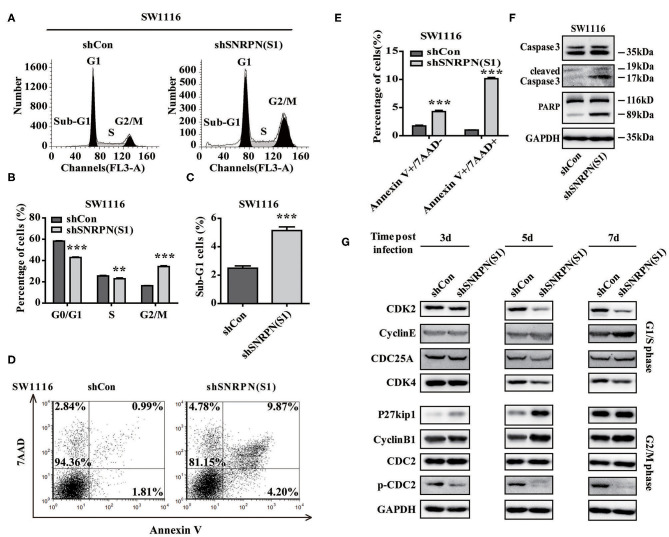
SNRPN suppresses cell cycle and promotes apoptosis. **(A)** Representative picture of the cell cycle assayed using flow cytometry with PI staining. **(B)** Histogram of cell number in G0/G1, S, and G2/M phases. ^**^P < 0.01; ^***^P < 0.001. **(C)** Histogram of cell number in the sub G1 phase. **(D)** Representative picture of apoptosis assayed using flow cytometry with 7-ADD and Annexin V double staining. **(E)** Percentage of apoptotic cells. **(F)** Western blotting analysis of apoptosis markers. **(G)** Western blotting analysis of markers involved in the cell cycle.

To examine whether downregulation of SNRPN affects the cell cycle, flow cytometry was performed using PI staining. The results showed a decreased number of cell in the G0/G1 and S phases and enrichment of cells in the G2/M phase compared to controls (Figures 5A,B). These results indicate an arrest in the G2/M phase transition after downregulation of SNRPN. In addition, the number of cells in the sub G1 phase increased, which suggests cell apoptosis after downregulation of SNRPN (Figure 5C). Apoptosis was detected using FCM with 7-aminoactinomycin D (7-ADD) and Annexin V double staining. The results show that the apoptosis rate was significantly increased, with 9.87% of apoptotic cells in the SNRPN downregulation group compared to only 0.99% in the control group (Figures 5D,E). In addition, Western blotting results show that the levels of cleaved caspase 3 and PAPR increased (Figure 5F). These results indicated that the absence of SNRPN results in apoptosis in CRC cells.

The mechanisms underlying the growth suppression by reduced SNRPN was investigated using Western blotting to determine the levels of molecular markers involved in the cell cycle 3, 5, and 7 days after transduction by lentivirus with shSNRPN. The results show that cyclin-dependent kinases CDK2 and CDK4 levels declined after 5 and 7 days, whereas cyclin E levels increased after 7 days, regulating the G1/S phase. In addition, P27kip1 levels were upregulated after 3 and 5 days, but restored after 7 days. The expression levels of another marker involved in the G2/M phase, CDC2, remained unaltered, although the levels of phosphorylated CDC2 decreased significantly (Figure 5G).

### Downregulation of SNRPN Impairs Growth of Human CRC Xenografts

**Figure 6:**
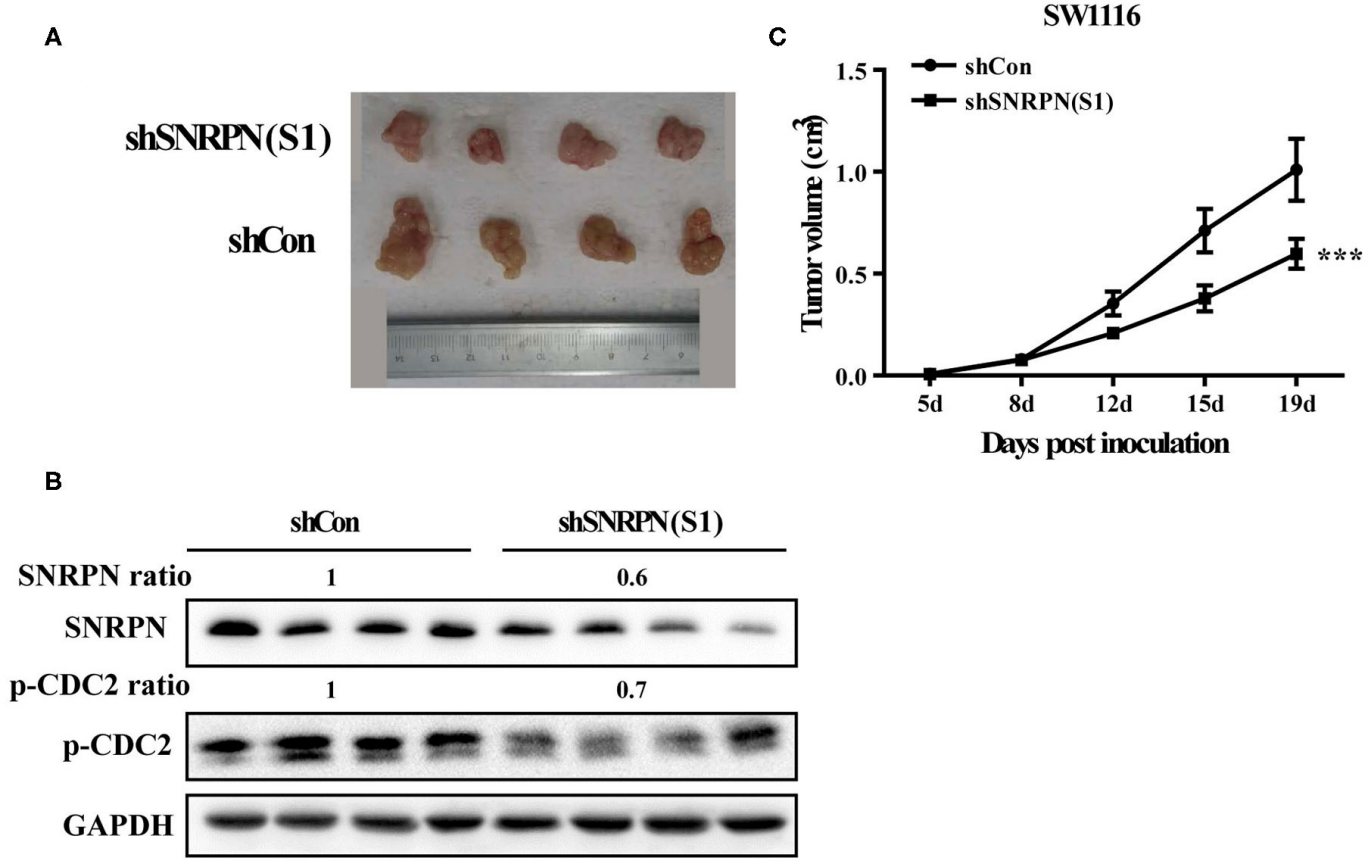
Downregulation of SNRPN impairs growth of human colorectal cancer xenografts. **(A)** Effect of SNRPN on tumor formation in a nude mouse xenograft model. Pictures of tumors were taken after sacrifice on day 19. **(B)** Western blotting analysis of SNRPN and p-CDC2 expression levels. **(C)** The tumor size was smaller compared with the control group measured twice a week from day 5 after injection. ^***^P < 0.001.

To determine the inhibition of cancer cell proliferation *in vivo*, we subcutaneously injected SW1116 cells transduced with shRNA(S1) and control cells into the left rear flank of Barb/c xenograft mice. Tumor size and body weight were measured twice a week. We found that low SNRPN expression significantly reduced tumorigenicity (Figure 6A). The tumor was found the first week after injection of cells, and its size was significantly smaller than that in the control group (Figure 6C). Xenograft tissue was collected after 19 days of growth. The assessment of SNRPN and the cell cycle marker p-CDC2 shows that downregulation of SNRPN reduced the levels of p-CDC2 and verified the cell cycle arrest *in vivo* (Figure 6B).

### E2F8 Is One of Transcriptional Regulators Up-Steam of SNRPN

**Figure 7:**
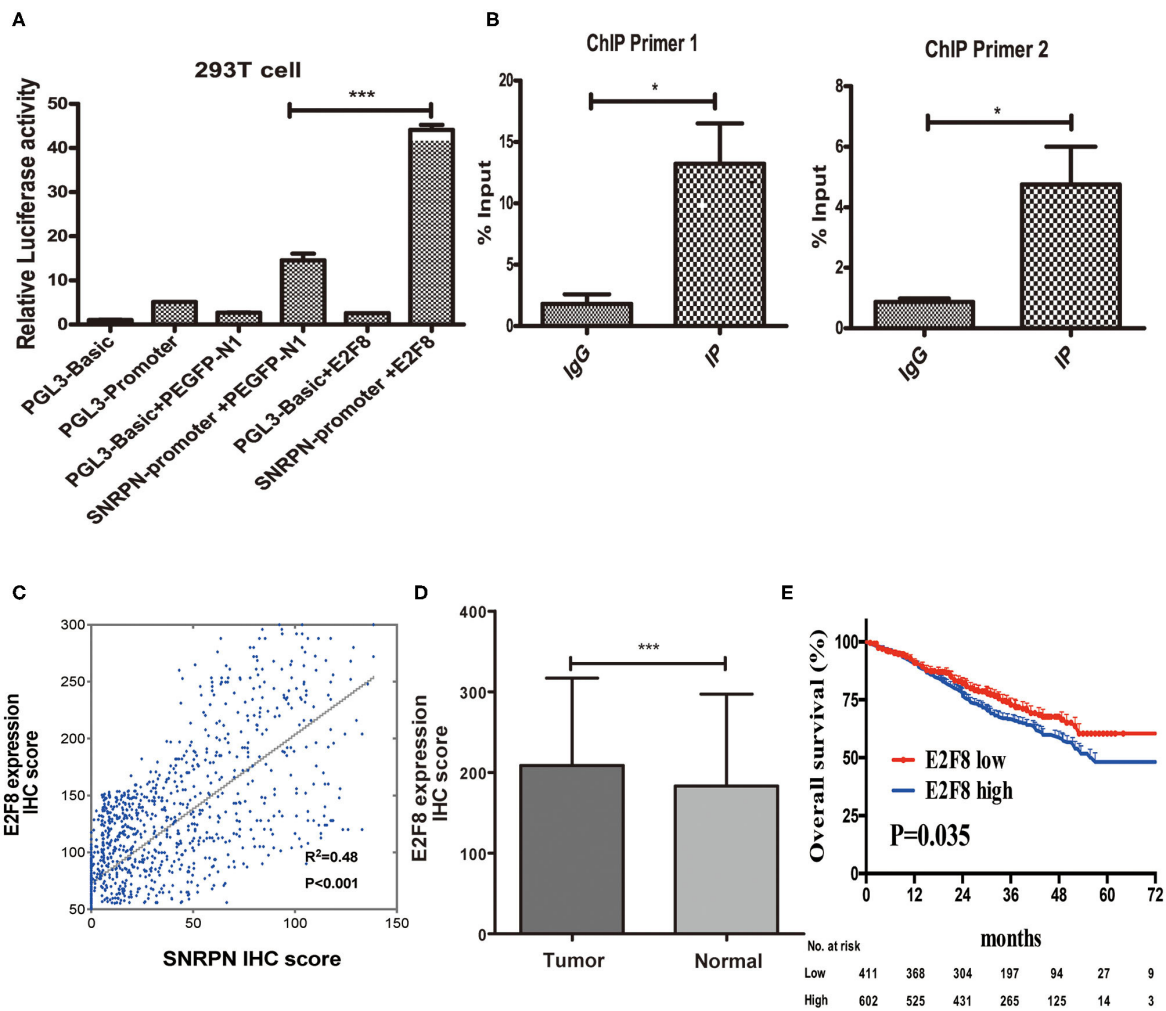
E2F8 is one of the transcriptional regulators up-steam of SNRPN. **(A)** Relative Luciferase activity of SNRPN promoter. **(B)** ChIP-qPCR assay for E2F8's occupancy on the SNRPN promoter. **(C)** Correlation analyses of E2F8 expression with expression of SNRPN using IHC staining including 1,310 colorectal cancer tissue samples. **(D)** IHC staining score of E2F8 was significantly higher in tumor tissues than in normal tissues. **(E)** Kaplan–Meier analysis of the association between E2F8 expression and overall survival. ^*^P < 0.05; ^***^P < 0.001.

Recently, researches showed us abnormal methylation is one of the most important mechanisms for SNRPN, yet, we did not find the correlation of abnormally high expression of SNRPN and its methylation level in our study. To figure out the cause of high expression of SNRPN in CRC, we experimented to find possible up-steam regulators. Firstly, we found the promotor activity sequence by promotor 2.0 and predicted its possible binding transcript factor by database JASPAR. We then conducted the plasmids for luciferase reporter assay, and the results showed that the activity of the SNRPN (2,500 bp) gene promoter was three times than that of its control group under the regulation of the E2F8 transcription factor (Figure 7A, *P* < 0.001). This indicates that the E2F8 transcription factor can promote the SNRPN gene promoter. To further verify its endogenous binding, ChIP experiment was performed, and ChIP-qPCR indicated that E2F8 was a transcriptional regulator up-steam of SNRPN (Figure 7B). Besides, we found a positive correlation between the expression of E2F8 and SNRPN in patient tissues by the same set of tissue microarray (Figure 7C). In addition, it showed that IHC scores of E2F8 in tumor tissues were significantly higher than those in normal tissues (Figure 7D). The association between E2F8 expression levels and clinical characteristics is shown in Supplementary Table 1. Analysis demonstrated that the expression of E2F8 was significantly associated with gender, carcinoembryonic antigen (CEA) level, tumor differentiation, pathological type, vascular invasion, neural invasion, and distant metastasis. Increased expression of E2F8 was significantly associated with unfavorable OS in CRC (Figure 7E).

## Discussion

SNRPN is an imprinted gene for paternal expression and participates in the regulation of cell differentiation, cell proliferation, embryonic development, and mental behavior. It usually binds to a precursor RNA and thereby translates into different peptides by modulating alternate RNA splicing (17, 18). Until now, no studies on the participation of SNRPN in CRC have been described. In the present study, we found that SNRPN exhibits significantly increased expression levels in CRC tissues compared to adjacent normal tissues. Additionally, clinical and pathological data show that increased SNRPN expression is correlated with larger tumor size, tumor location on the left-sided colon, neural invasion, and distant metastasis. Moreover, patients with higher SNRPN expression levels had a poorer overall survival rate. These results indicate that the abnormal expression of SNRPN is associated with the development and progression of CRC. Further exploration of SNRPN biological function in CRC cells confirmed that the abnormal expression of SNRPN promotes the proliferation and metastasis of CRC cells and tumorigenicity.

Malignant proliferation is one of the hallmarks of cancer, and cell cycle plays an important role. Cell cycle progression depends on the activation of CDKs. In this study, we observed a time-dependent decrease in CDK4 and CDK2 levels and an increase in the levels of cyclin E, involved in the G0/G1 phase transition, after downregulation of SNRPN. In addition, an increase in cell arrest at the G2/M phase resulted from upregulating P27Kip1, cyclin B1, and especially inactivity of CDC2. CDC2 plays a critical role in cell cycle inducing the G2/M transition and anti-mitosis. CDC2 is inactive in phosphorylated form, which induces the inactivation of the cyclin B/CDC2 complex and results in G2/M arrest (19, 20). Our study shows that SNRPN downregulation contributed to reduced CDC2 activity, ultimately leading to a G2/M cell cycle delay. In addition, an increase in the number of cells in the sub G1 phase indicates that SNRPN downregulation exhibits a strong anti-proliferative effect on CRC cells by promoting cell apoptosis.

Genomic imprinting is one important epigenetic phenomenon during gametogenesis, which accompanied DNA methylation of genes or gene clusters. This plays a major role in mammalian development, such as maintenance of chromatin structure and cell differentiation. CpG methylation is a central mechanism of epigenetic gene regulation and maintaining the imprinted state (21). Structurally, the SNRPN gene has 23 CpG islets. Methylation is therefore an important mechanism of regulating SNRPN biological function.

SNRPN hyper-methylation was observed in some diseases, 34.9% in myelodysplastic syndrome and 50% in acute myeloid leukemia (22). The methylation levels of SNRPN revealed statistical differences between adults and children, which indicates the role of SNRPN during the aging process (23). Differential methylation patterns of SNRPN have also been reported in multiple types of cancer. SNRPN methylation patterns in germ cell tumors reflect the primordial germ cell development (24). In gastric cancer, SNRPN acts as a novel hyper-methylated gene with corresponding reduced expression levels compared with intestinal metaplasia. In our study, the correlation of methylation and SNRPN expression levels in CRC had not been verified by TCGA data assay, but whether the high expression of SNRPN in CRC is mediated by abnormal methylation needs to be further explored.

To figure out the cause of high expression of SNRPN in CRC, we explored the up-steam molecular regulator. Luciferase reporter assay and ChIP assay were employed to identify interactor of SNRPN; interestingly, we found E2F8 was one of the transcriptional regulators of SNRPN. E2F8 is a family member of the E2f transcription factors. It plays a crucial role in control of cell proliferation, differentiation, apoptosis, and gluconeogenesis (25, 26). Previous studies identified that the most significant biological process of E2F8 was regulating cell cycle (27, 28). Moreover, it was abnormally expressed in different kinds of cancers, such as hepatocellular carcinoma, lung cancer, cervical cancer, and papillary thyroid cancer. The mechanism was defined as oncogene by influencing cell cycle (29–32).

In CRC, E2F8 was highly expressed in cancer tissues and cell lines and a direct target of miR-1258 regulating cell cycle genes (33). Knockdown of E2F8 suppresses cell proliferation in colon cancer cells by modulating the NF-KB pathway (34). E2F8 acted as a stemness gene marker and was upregulated after celecoxib treatment in HT29 and DLD1 cells (35). In our study, we demonstrated that SNRPN was a target gene of E2F8, and increased expression of E2F8 induces high expression of SNRPN to regulate the malignant proliferation of cells by cell cycle regulation.

In conclusion, SNRPN was abnormally highly expressed in CRC tissues compared to adjacent normal tissues in 1,310 clinical samples, and its high expression was negatively associated with overall survival. Besides that, the loss of SNRPN expression in CRC cells decreased cell growth and metastasis, inhibited cell proliferation regulated by the cell cycle, and promoted apoptosis. The present study provides important evidence of the axis of E2F8/SNRPN in colorectal cancer.

## Data Availability Statement

The raw data supporting the conclusions of this article will be made available by the authors, without undue reservation.

## Ethics Statement

The studies involving human participants were reviewed and approved by Clinical Research Ethics Committee of Zhongshan Hospital, Fudan University. The patients/participants provided their written informed consent to participate in this study. The animal study was reviewed and approved by Animal Ethics Committee of Zhongshan Hospital Fudan University.

## Author Contributions

MJ wrote the paper and completed the experiment. XL and JX designed the study. LR, YL, and QF contributed to the collection and analysis of clinical data. WT, AZ, TL, and PZ followed-up clinical data. All authors contributed to the article and approved the submitted version.

## Conflict of Interest

The authors declare that the research was conducted in the absence of any commercial or financial relationships that could be construed as a potential conflict of interest.
