# Supplementary material for: Small Nuclear Ribonucleoprotein Polypeptide N Accelerates Malignant Progression and Poor Prognosis in Colorectal Cancer Transcriptionally Regulated by E2F8
Source: Front Oncol. 2020 Nov 2;10:561287. doi: 10.3389/fonc.2020.561287 (PMC7669248; doi:10.3389/fonc.2020.561287)
Supplement: Supplementary file 1 [file Table_1.DOCX]

|  | **Total** | **E2F8 expression** | | |
| --- | --- | --- | --- | --- |
| **Factors** |  | **Low** | **High** | **P-value** |
| **All patients** | 1013 | 411 | 602 |  |
| **Age (years)** |  |  |  | 0.345 |
| ≥60 | 576 (56.9%) | 241 (58.6%) | 335 (55.6%) |  |
| <60 | 437 (43.1%) | 170 (41.4%) | 267 (44.4%) |  |
| **Gender** |  |  |  | 0.000 |
| Male | 594 (60.6%) | 293(71.3%) | 301(50.0%) |  |
| Female | 419 (40.0%) | 118 (28.7%) | 301(50.0%) |  |
| **CEA (ng/ml)** |  |  |  | 0.002 |
| ≤ 5 | 523 (53.2%) | 211(51.3%) | 222(55.1%) |  |
| > 5 | 460 (43.1%) | 193(47.0%) | 158(39.2%) |  |
| Unknown | 30 (3.7%) | 7 (1.7%) | 23 (5.7%) |  |
| **Tumor size** |  |  |  | 0.894 |
| ≤5cm | 387 (38.2%) | 156(40.3%) | 231(40.7%) |  |
| >5cm | 626 (61.8%) | 255(59.7%) | 371(59.3%) |  |
| **Tumor differentiation** |  |  |  | 0.000 |
| Well/Moderately | 684 (67.5%) | 364 (88.6%) | 320 (53.2%) |  |
| Poorly/anaplastic | 329 (32.5%) | 47 (11.4%) | 282 (46.8%) |  |
| **Pathological type** |  |  |  | 0.000 |
| Mucinous | 162 (16.0%) | 89 (21.7%) | 73 (12.1%) |  |
| Non-mucinous | 851 (84.0%) | 322 (78.3%) | 529 (87.9%) |  |
| **Vascular invasion** |  |  |  | 0.000 |
| No | 827 (81.6%) | 305(74.2%) | 522 (86.7%) |  |
| Yes | 186 (18.4%) | 106(25.8%) | 80 (13.3%) |  |
| **Neural invasion** |  |  |  | 0.000 |
| No | 760 (75.0%) | 241(58.6%) | 519 (86.2%) |  |
| Yes | 253 (25.0%) | 170 (41.4%) | 83 (13.8%) |  |
| **Tumor location** |  |  |  | 0.142 |
| Right-sided colon | 290 (27.8%) | 110 (26.8%) | 180 (29.9%) |  |
| Left-sided colon | 263 (26.0%) | 99 (24.1%) | 164 (27.2%) |  |
| Rectum | 460 (46.2%) | 202 (49.1%) | 258 (42.9%) |  |
| **T stage** |  |  |  | 0.517 |
| T1/T2 | 173 (18.1%) | 74 (18.0%) | 99 (16,4%) |  |
| T3/T4 | 840 (81.9%) | 337 (82.0%) | 503(83.6%) |  |
| N stage |  |  |  | 0.742 |
| N0 | 583 (54.9%) | 234 (56.9%) | 349 (58.0%) |  |
| N1/N2 | 430 (45.1%) | 177 (43.1%) | 253 (42.0%) |  |
| **M stage** |  |  |  | 0.000 |
| M0 | 729 (76.7%) | 324(78.8%) | 405 (67.3%) |  |
| M1 | 284 (23.3%) | 87 (21.2%) | 197 (32.7%) |  |

Table: Relationship between E2F8 and clinical characteristics in included CRC patient
